# The Role of Diet and Specific Nutrients during the COVID-19 Pandemic: What Have We Learned over the Last Three Years?

**DOI:** 10.3390/ijerph20075400

**Published:** 2023-04-04

**Authors:** Petra Rust, Cem Ekmekcioglu

**Affiliations:** 1 Department of Nutritional Sciences, University of Vienna, Josef-Holaubek-Platz 2, 1090 Vienna, Austria; 2 Department of Environmental Health, Center for Public Health, Medical University of Vienna, 1090 Vienna, Austria

**Keywords:** COVID-19, SARS-CoV-2, infection risk, disease severity, nutrition, diet, micronutrients, vitamin D, vitamin C, zinc, selenium, omega-3 fatty acids

## Abstract

Nutrients and diets have an important impact on our immune system and infection risk and a huge number of papers have been published dealing with various aspects of nutrition in relation to SARS-CoV-2 infection risk or COVID-19 severity. This narrative review aims to give an update on this association and tries to summarize some of the most important findings after three years of pandemic. The analysis of major studies and systematic reviews leads to the conclusion that a healthy plant-based diet reduces the risks for SARS-CoV-2 infection and especially COVID-19 severity. Regarding micronutrients, vitamin D is to the fore, but also zinc, vitamin C and, to some extent, selenium may play a role in COVID-19. Furthermore, omega-3-fatty acids with their anti-inflammatory effects also deserve attention. Therefore, a major aim of societal nutritional efforts in future should be to foster a high quality plant-based diet, which not only exerts beneficial effects on the immune system but also reduces the risk for non-communicable diseases such as type 2 diabetes or obesity which are also primary risk factors for worse COVID-19 outcomes. Another aim should be to focus on a good supply of critical immune-effective nutrients, such as vitamin D and zinc.

## 1. Introduction

Since December 2019, the world has been confronted with the outbreak of the coronavirus disease 2019 (COVID-19) pandemic, caused by the severe acute respiratory syndrome coronavirus 2 (SARS-CoV-2) [1,2]. The symptoms of COVID-19 are diverse, notably including fever, cough, shortness of breath, myalgia or fatigue [1,3,4] but also, for example, new loss of taste and smell or diarrhea [5]. Critically ill patients may suffer from abnormal coagulation and excessive inflammation leading, for example, to acute respiratory distress syndrome (ARDS) or cardiac injury [1,4]. Therefore, in addition to the respiratory tract, other organ systems, such as the gastrointestinal tract [6] and the cardiovascular system [7], may be involved in the disease.

Healthy nutrition including an adequate supply of essential and bioactive nutrients is not only vital for human health and well-being but also has an important protective impact for respiratory infectious diseases such as COVID-19 [8,9]. In this regard, nutrition addressing the SARS-CoV-2 pandemic, but also other (respiratory) virus crises which could challenge mankind in the future, could/should fulfill following criteria:

- support the immune system;
- show beneficial effects on inflammation and therefore possibly reduce the risk of an excessive inflammatory response leading to tissue damage;
- improve protection against oxidative stress;
- reduce the risk of SARS-CoV-2 infection and/or protect against severe COVID-19 disease progression;
- in the medium to long term, reduce the risk of diseases, such as diabetes and obesity, among others, that are associated with a weakened immune system and poorer COVID-19 “outcomes”.

Various factors besides healthy nutrition and various nutrients (see below) influence the efficiency of the immune system and the immune response. These include some unmodifiable factors such as genetics, life stage, and also—for example—time of day. However, most of the factors, such as vaccination, stress, physical fitness, frailty, medication, body fatness, smoking, alcohol consumption and also diet are modifiable [10]. 

Aging is the most important unmodifiable factor and is associated with various physiological impairments of especially the acquired but also the innate immune system [11,12]. This age-related alteration is termed immunosenescence [13], which in combination with inflammaging, an increased inflammatory state, might contribute to infection severity [14]. In this regard, older persons have higher risks for severe forms of disease, poorer outcomes during hospitalization, and of death from COVID-19 [12,15]. Respiratory infection is one of the most important reasons for mortality in the elderly [16] and a lack of certain micronutrients may increase the risk of respiratory infections.

From the modifiable factors, body fatness has a prominent role. In obesity the immune system is weakened due to factors such as chronic inflammation, changes in the T-cell profile, and accompanying diseases such as diabetes. Furthermore, obese individuals show significantly higher risks for morbidity and mortality from COVID-19 [10,17,18]. Inflammation in the adipose tissue, immune impairments and metabolic dysfunctions but also a higher affinity of SARS-CoV-2 for the angiotensin-converting enzyme 2 (ACE2) receptor, highly expressed in adipose tissue [17,19,20,21,22], are considered to play a role in this higher risk.

COVID-19 is associated with an inflammatory state and especially patients with severe disease courses in intensive care units (ICU) exhibit elevated plasma levels of several cytokines, declared as a “cytokine storm”, leading to excessive inflammation [23,24]. Oxidative stress might also play a role in COVID-19 pathogenesis and disease severity [25,26]. In this regard, variations in redox homeostasis, impairments in antioxidant defenses, chronically elevated levels of reactive oxygen species (ROS) and induction of ROS-generating enzymes are central factors linking infections with respiratory viruses, including influenza virus and SARS-CoV [27,28]. Prolonged oxidative stress occurs in chronic viral infections, as with the Epstein–Barr virus (EBV) and the human immunodeficiency virus (HIV), among others [29,30] and has been associated with impaired immune responses [31,32].

Nutrients and diets have an important impact on our immune system and infection risk and in the last three years a large number of papers have addressed various aspects of nutrition in relation to SARS-CoV-2 infection risk or COVID-19 severity. This narrative review therefore intends to give an update on this association and summarizes some of the most important findings.

## 2. Diet Recommendations, Diet Quality and COVID-19

Consuming good quality diets is essential for a healthy immune system, and deficiencies of numerous micronutrients increase a person’s susceptibility to virus infection and also the risk for severe clinical presentation [33]. In the context of the SARS-CoV-2 crisis, various renowned institutions around the world have issued dietary recommendations (summarized in [34]). Most nutrition societies, health organizations such as the WHO or UNICEF, as well as national organizations recommend the consumption of vegetables, fruits and whole grains, as main components of a healthy diet. Diets rich in vegetables and fruits contain especially vitamin C, beta-carotene, folate, zinc, and selenium which are important for the immune system. In addition, these food groups provide many health-promoting polyphenols that exert anti-inflammatory, antioxidant and partially anti-viral effects [35]. For example, a systematic review and meta-analysis showed that flavonoid supplementation in the range of 0.2 to 1.2 g/day in 14 selected studies decreased the incidence of upper respiratory tract infections by 33% compared with control [36].

Dietary fiber consumption is also relevant. For example a higher fiber consumption is associated with a lower risk of death from cardiovascular, infectious, and respiratory diseases [37]. For every 10 g/day increase in dietary fiber intake, the multivariate RRs for death from infectious and respiratory diseases were 0.66 (95% CI: 0.52–0.84) and 0.82 (95% CI: 0.74–0.93), respectively, in men and 0.61 (95% CI: 0.44–0.85) and 0.66 (95% CI: 0.56–0.78), respectively, in women. Furthermore, in a recent umbrella review of meta-analyses published in the BMJ [38] it was shown that a higher intake of whole grains and cereal fiber significantly reduced the risk of type 2 diabetes, with a high quality of evidence. 

Vajargah et al. observed positive effects in COVID-19 patients also. Study participants who consumed more fruits, vegetables and fiber were less likely to develop severe COVID-19, required less corticosteroids and antiviral medication, and had lower inflammatory markers (i.e., CRP) and a significantly shorter length of hospital stay and convalescence [39]. Dysbiosis triggered by binding of SARS-CoV-2 to ACE2 receptors on the surface of enterocytes or by circulating cytokines could cause a leaky gut, which in turn favors the entry of bacterial products and toxins [40]. Studies show severe gastrointestinal symptoms and higher stool calprotectin levels, indicating gastrointestinal inflammation, in COVID-19 patients [41,42]. 

In addition to vegetables, fruit and whole-grain products, legumes, nuts and seeds, as well as fish as a source of omega-3 fatty acids, and low-fat (fermented) dairy products should complement the diet. These food groups ensure a good supply of protein, health-promoting fatty acids and calcium. In addition, fermented dairy products support good intestinal flora. The consumption of meat should be kept to a minimum to minimize the intake of saturated fatty acids. In consumption, preference should also be given to fresh food over highly processed food.

It is important to emphasize that no single food or food group alone can prevent or cure COVID-19 infection. It is therefore important to adapt eating patterns so that the consumption of plant-based foods predominates and the diversity in the individual food groups is also taken advantage of. By consuming a balanced and varied diet, nutrient deficiencies that increase the risk of severe COVID-19 infection can be prevented.

### 2.1. Plant-Based Diets and COVID-19

According to the WHO “Plant-based diets constitute a diverse range of dietary patterns that emphasize foods derived from plant sources coupled with lower consumption or exclusion of animal products.” [43]. A healthful plant-based diet (PBD) prioritizes fruits, vegetables, whole grains, legumes and nuts, while an unhealthful PBD includes in relevant amounts fruit juices, sweets, sugar sweetened beverages and refined grains [44]. In this review PBD are considered to be healthful, if not otherwise mentioned. While not strictly plant-based, the Mediterranean diet is also considered in this category [45].

Several recent important papers have shown that healthy PBDs can reduce the risk for SARS-CoV-2 infection and especially are able to reduce the risk for severe COVID-19. For example, in a case-controlled study of healthcare workers, primarily physicians, across six countries, who were highly exposed to COVID-19 patients, Kim et al. found a 72% or 59% reduction in the odds ratio of severe COVID-19 in those consuming either a PBD or PBD/pescatarian diet, respectively [46]. On the other hand, compared with participants who followed a PBD, those reporting low carbohydrate and high protein diets had nearly 4 times higher odds of moderate-to-severe COVID-19. The study was not influenced by patients’ vaccine status.

Another well published study which used data from the smartphone-based COVID-19 Symptom Study with nearly 600,000 participants recruited in the prevaccination period in 2020 found that those who had a high healthful PBD index score were 41% less likely to have severe COVID-19 symptoms and showed 18% lower risk for positive tested SARS-CoV-2 infection compared to those who had a low healthful PBD score [8]. The association of the PBD with COVID-19 was suggested to be particularly evident among individuals with high socioeconomic deprivation [8]. In this regard, the socioeconomic status, and in particular education and income levels, are associated with food choice and influence micronutrient intake and status [47,48]. Higher educational status leads to better nutrient intake, especially in countries with lower gross domestic product (GDP). For example, a 10% higher GDP was associated with lower total fat intakes and higher daily total folate intakes in higher educated individuals from 12 European countries [49].

Furthermore, in a Polish study in non-obese healthy physically active younger adults it was shown that those with an average daily consumption of >500 g of vegetables and fruit and >10 g of nuts had an 86% lower risk of COVID-19 compared to those following a non-balanced diet, who consumed lower amounts of these food groups and who showed a higher dietary inflammation index [50].

Plant foods are, among others, a good source of folate, vitamin C, vitamin K and fiber. A recent French study calculated, in addition to a higher fruit and vegetables consumption, that all of these four nutrients were associated with a decreased probability of SARS-CoV-2 infection, while on the other hand a higher odds ratio was calculated for dairy products and calcium intake [51]. Moreover, an ecological study of 158 countries across the globe calculated a significant positive correlation between the crude SARS-CoV-2 infection rate and calcium or total milk intake [52].

Another study in 8801 adults from Iran showed that a higher intake of high-fat-dairy-products or, for example, cheese was related to a 40 or 80% increase in the odds of COVID-19, respectively; on the other hand, a moderate intake of total dairy products and also use of low-fat dairy products showed a protective effect [53]. A high intake of long chain saturated fatty acids might be detrimental regarding COVID-19 risk because it might, for example, increase the population of harmful bacteria in the gut and the production of highly inflammatory endotoxin molecules [54]. The beneficial effects of PBD could therefore also be due to their lower content of long chain saturated fatty acids, which have been calculated to have a highly positive pro-inflammatory effect score [55]. 

The Mediterranean Diet (MeD) also shows beneficial effects in the protection against COVID-19 [56]. According to previous meta-analyses, adherence to the MeD reduces the risk for several diseases, including some cancers, cardiovascular pathologies and metabolic disease [57]. Relevant for the current review are previous observations suggesting a reduced risk for respiratory infections with the MeD [58] and especially a reduced risk of inflammation, with a decrease in C-reactive protein (CRP) and pro-inflammatory cytokines, such as interleukin (IL)-6 and IL-1β [59,60].

In a recent study in 5194 non-health professionals, participants with intermediate and highest adherence to the MeD had significantly lower odds of developing COVID-19 (multivariable-adjusted OR = 0.50, 95% CI: 0.34–0.73 for intermediate and OR = 0.36, 95% CI: 0.16–0.84 for high adherence) compared to those with a low adherence [56].

Likewise, in two large US cohorts, high adherence to a Mediterranean style diet was associated with a 22% lower likelihood of SARS-CoV-2 infection compared to low adherence [61]. Participants with a healthier diet additionally showed a lower odds ratio for severe infection and hospitalization. However, results were no longer significant after controlling for BMI and pre-existing medical conditions [61].

In summary, it can be concluded that an adequate supply of micronutrients—achieved through a balanced diet high in vegetables, legumes and fruits—is important for the integrity of the physical barrier, e.g., in the gastrointestinal or respiratory tract, and for the functioning of the innate and adaptive immune system as well as its antioxidant potential. The mechanisms underlying the positive effects are discussed in the third part of this review covering single nutrients. Furthermore, the content of dietary fiber and probiotics of wholegrain and fermented low-fat dairy products in the diet is relevant to improving the microbiota, which in turn have a positive effect on the immune system. In addition, the quality of fat in the diet plays an important role in influencing the immune system: while a high consumption of long-chain saturated fatty acids promotes low-grade inflammation, monounsaturated and polyunsaturated fatty acids, especially omega-3 fatty acids, have a favorable immune-modulatory effect. It is therefore recommended to reduce meat consumption, while increasing the intake of fish, nuts, seeds, and high-quality oils [62]. Last but not least, too much salt as well as sugar-sweetened and high-fat products should be avoided, as they contribute to increasing the risk of obesity, a risk factor for COVID-19 [63].

### 2.2. Western Type Diet and Ultra-Processed Food Intake

In contrast to a good quality diet, the Western type diet (WeD) is characterized by a high supply of sugar, salt, white flour, and saturated animal fat, with little intake of antioxidants and fiber [64]. In addition, WeDs are particularly energy-dense and exhibit high glycemic indexes. Various studies have shown that such an unbalanced diet is associated with an increased risk for type 2 diabetes [65], and for chronic inflammation [66]. In detail, there is a repeated association between dietary intake of long chain saturated fatty acids and inflammation markers and chronic systemic low-grade inflammation [62,67,68]. Low-grade chronic inflammation, which is especially found in patients with obesity and diabetes mellitus, has been proposed to favor the cytokine storm, which is associated with SARS-CoV-2 infection severity [69,70]. 

In a recent preprint, the detrimental effects of a high-fat high-sugar WeD on COVID-19 outcome were shown in Syrian hamsters. The authors observed increased weight loss and lung pathology, and also, delayed viral clearance and functional lung recovery in the animals studied, together with prolonged viral shedding [71].

Ultra-processed foods (UPF), such as breakfast cereals, savory snacks, reconstituted meat products, frankfurters, pre-packaged frozen dishes, soft and/or sweetened drinks [72] also belong to an unhealthy dietary pattern, especially when consumed in large quantities. An increased intake of UPF is not only associated with a higher risk for overweight/obesity, cardio- and cerebrovascular diseases, depression and all-cause mortality [73], but also for COVID-19 [74]. In 41,012 participants from the UK Biobank study it was shown that, compared to participants in the lowest quartile of UPF consumption those in the highest quartile, were associated with a 22% higher probability of COVID-19, after adjusting for potential confounders [74]. This association was partly mediated by the BMI and showed a nonlinear course, with flattening after around 30% of the predicted proportion of UPF consumption. Reasons for the adverse effects of UPF intake on COVID-19 might be pro-inflammatory effects due to the presence of excess amounts of simple sugars and saturated fatty acids [75] and a low intake of immunoprotective vegetables and fruits; moreover, essential micronutrients could be at least partly lost during food processing. 

## 3. Selected Nutrients and COVID-19

The third part of the review focuses on the background and mechanisms of action of selected nutrients. In addition to a healthy diet, some organizations also emphasize the importance of micronutrients such as zinc, vitamins D, C and A for the immune system and suggest that people and patients at risk, as well as people with deficiencies, should supplement certain micronutrients [34]. Extensive nutritional recommendations for COVID-19 patients were also published [76].

### 3.1. Vitamin D

The role of vitamin D regarding the regulation of calcium and phosphate metabolism and bone mineralization is well known. Apart from this, antibacterial effects of vitamin D have also been described [77]. However, vitamin D can also activate signaling cascades that promote antiviral innate immunity [77], which might play an important role in COVID-19 infection [78]. Most immune cells including monocytes/macrophages, dendritic cells, T and B cells express the receptor for vitamin D (VDR) and the enzyme 25-(OH)-D-1α-hydroxylase (CYP27B1) that transforms 25-hydroxyvitamin D (25(OH)D) into active vitamin D (1,25(OH)_2_D) [79]. All these functions are of interest for the prevention and therapy of infectious diseases. 

Various meta-analyses of intervention studies as well as “umbrella” reviews summarizing meta-analyses showed that vitamin D supplementation reduces the risk of respiratory tract infections [80,81,82]. Martineau et al., for example, noted that the benefit of Vitamin D supplementation was stronger in people receiving daily or weekly vitamin D (without an additional bolus dose) who had a very low vitamin D status at the beginning of the study (<25 nmol/L) with a (adjusted) risk reduction of up to 70% [82]. 

A large number of papers have been published in the last 3 years regarding 25(OH)D-status or vitamin D supplementation and SARS-CoV-2 infection risk or COVID-19 severity. Using the simple search term “vitamin D” and (COVID-19 or SARS-CoV-2) in PUBMED on the 20 January 2023 showed nearly 1500 papers and restricting the search to meta-analyses (by adding “and meta-analysis” to the search term) lists the considerable number of 73 hits. The major meta-analytical results and/or conclusions of the selected publications are summarized in Table 1.

For example, in a meta-analysis, Kazemi and researchers surveyed 39 retrospective and prospective cohort, cross-sectional, case–control, and randomized controlled trial studies (up to 26 November 2020) to assess the relation between 25(OH)D status and SARS-CoV-2 infection as well as COVID-19 severity [97]. In reports that were adjusted and non-adjusted for confounders, the researchers found a greater risk of SARS-CoV-2 infection in the vitamin D deficient group. Other more recent meta-analyses also consistently found a greater risk for COVID-19 in individuals with a lower vitamin D status [86,100,101,102,103,106,109,112]. 

Regarding the risk for severe COVID-19 and 25(OH)D status, one of the first systematic reviews from Pereira et al., which included studies up to 9 October 2020, found that severe cases of COVID-19 showed more vitamin D deficiency compared with mild cases (OR = 1.64; 95% CI: 1.30–2.09) [96]. In an addendum/update of this meta-analysis, the authors recalculated the data after excluding withdrawn “fake studies”, and showed that their conclusions had not changed. A general problem of the enormous study flood beginning from early Spring 2020 might be the risk for “pollution” of the scientific databases with COVID-19 studies with low quality and false results. This might be especially a problem in systematic reviews, whose authors should carefully check the included studies and update their reviews accordingly, as was the case in Pereira et al. [96] and Crafa et al. [105]. 

A further problem in systematic reviews (not only) investigating COVID-19 is combining study designs in meta-analyses with unadjusted data, which could lead to confounded findings [116]. Interestingly, in our summary of meta-analyses there were some considerable differences in, for example, selected papers or OR in meta-analyses which had a nearly similar date of search (Table 1). Reasons for these discrepancies could be different selection criteria, protocols and analyses [116].

Nevertheless, in addition to the results by Pereira et al., more recent meta-analyses also found consistently that a low vitamin D status/deficiency increases the risk for a more severe COVID-19 course and/or ICU admission [86,98,100,101,102,104,105,106,108,109,112]. 

In a meta-analysis by Dissanayake et al. it was shown that patients with severe COVID-19 had a 4.84 ng/mL mean lower 25(OH)D concentration compared to those without adverse outcomes [112]. However, it is still not definitely proven whether a low vitamin D status is a risk factor for COVID-19 and other inflammatory diseases or a consequence of the disease. Inflammation might reduce the 25(OH)D concentration or in the other direction a good vitamin D status might reduce inflammation (reviewed in [117]). Dynamic studies analyzing 25(OH)D levels at baseline and during the disease are necessary to gain more information regarding this controversial topic. In this context, it should also be kept in mind that low serum vitamin D levels are more common in people of older age and with chronic diseases [118].

Regarding vitamin D supplementation, two large primary prevention trials published in 2022 in the BMJ [119,120] did not show that daily supplementation for six months with either vitamin D at doses of 3200 IU or 800 IU [119] or as 5 mL cod liver oil (400 IU vitamin D) [120] reduced the risk for SARS-CoV-2 infection.

In contrast to preventive lower dose supplementation, clinical intervention trials with higher doses of vitamin D in hospitalized COVID-19 patients revealed some positive meta-analytical evidence especially on the risk for ICU admission [83,85,91,92,93] although not all meta-analyses showed significant beneficial effects [87,89] (Table 1). In addition, it is suggested that COVID-19 patients may rather benefit from receiving a daily or maintained in time vitamin D dose in contrast to a single vitamin D dose, which does not seem to have any effect on the health status of the patients studied [121,122].

The potential benefits of vitamin D in COVID-19 could be partly due to its anti-inflammatory and antioxidative effects with a recent umbrella meta-analysis including 23 meta-analyses with 21,148 participants, showing that supplementation with vitamin D led to reduced levels of CRP, TNF-alpha and malondialdehyde [123].

Nevertheless, although the effects of vitamin D regarding the innate and adaptive immune response are undisputed [124], further studies on dosage, effects of baseline vitamin D levels, and period of supplementation, and on safety are needed before a routine application of vitamin D for the management of COVID-19 can be recommended [84,125].

### 3.2. Vitamin C

Ascorbic acid has several essential physiological functions. It acts as an enzyme cofactor and antioxidant, strengthens the epithelial barrier and contributes to wound healing. In addition, vitamin C provides protection against ROS-induced damage and supports the innate and adaptive immune system [126,127].

The protection of immune cells from oxidative damage during the respiratory burst could explain the high levels of vitamin C in immune cells [128,129]. A deficiency of vitamin C results in impaired phagocytosis and respiratory burst, with supplementation of this antioxidant reversing these negative effects (reviewed in [130]). In this regard, a low vitamin C status indicates increased susceptibility to infections such as pneumonia, possibly because increased oxidative stress cannot be averted [131]. In a Cochrane review it has been stated that, due to low risks, therapeutic vitamin C supplementation may be reasonable for patients with pneumonia who have low vitamin C plasma levels [132].

Although some functions of vitamin C suggest a beneficial effect of vitamin C supplementation (0.25 to 2 g/day) on incidence and severity of colds, this has not been confirmed in meta-analyses in the general population [133]. However, some trials that investigated regular vitamin C supplementation showed reduced common cold incidence in individuals experiencing short periods of extreme physical stress and also benefits regarding the duration of colds, with greater advantages in children compared to adults [127,133]. In the CITRIS-ALI randomized clinical trial it was found that a 96-h infusion of vitamin C (50 mg/kg in dextrose 5% in water every 6 h for 96 h) could not modify levels of CRP, thrombomodulin, and modified Sequential Organ Failure Assessment (mSOFA) scores in patients with sepsis and severe acute respiratory distress syndrome. However, compared to the placebo group the mortality rate was significantly lower by 16.5%, and ICU-free days and hospital length of stay by 3.2 and 6.7 days, respectively [134]. Since these results are based on analyses that did not consider multiple comparisons, further studies considering higher dosages and longer duration of supplementation are needed to provide treatment recommendations for patients with sepsis and ARDS. No unexpected adverse effects occurred [134].

The evidence that vitamin C inhibits reproduction of viruses such as influenza type A, Herpes simplex virus type I and poliovirus type 1, and can shorten the duration of respiratory virus infection independent from the respiratory viruses leads to the suggestion that vitamin C is also important with respect to SARS-CoV-2 [135].

For example, in a Swiss cohort it was shown that COVID-19 patients had significantly lower plasma ascorbate levels than the controls, and stratification by disease showed significant differences in total ascorbate levels between healthy controls (median = 46.7 μM) and mild (median = 10.2 µM), severe (median = 2.8 µM), critical (median = 2.0 µM), and fatal (median = 1.8 µM) COVID-19 cases [136]. This was confirmed in a study from Spain showing that up to 82% of critically ill COVID-19 patients had low vitamin C levels (<23 µmol/L) [137].

Since vitamin C deficiency exhibits adverse effects in terms of immune function and organ damage, it is assumed that supplementation with high doses of vitamin C might support the treatment of critically ill COVID-19 patients [138].

Thomas et al. [139], for example, compared effects of 10 days orally administered high-dose zinc (50 mg zinc gluconate/d), high-dose ascorbic acid (8 g/d divided on 2–3 doses), and/or a combination of the two, and usual care on the duration of symptoms of 214 eligible participants with SARS-CoV-2 infection. A non-significant 50% reduction in symptoms of 1.2 d for the ascorbic acid group, 0.8 d for the zinc gluconate group, and 1.2 d for the group receiving both micronutrients was observed compared to the usual care group [139]. Hemilä et al. stated that the variation in the duration of untreated SARS-CoV-2 infection ranges from 2 days to 3 weeks, which means that the mean difference is not very suitable to measure treatment effects. In a recalculation of the data, the authors found that vitamin C increased the rate of recovery by 70% [140].

Zhang et al. observed in a randomized controlled pilot trial performed in 56 critical COVID-19 patients in three Chinese hospitals that there were no differences in invasive mechanical ventilation-free days in 28 days between the intervention group which received 12 g of vitamin C/50 mL every 12 h for 7 days and the placebo group. However, pulmonary function improved and IL-6 declined showing that vitamin C inhibited production and release of pro-inflammatory cytokines. Due to the small sample size and the late initiation of vitamin C treatment (more than 10 days after the first symptoms), the results are not conclusive [141]. In another study in 85 patients with severe COVID-19, high dose intravenous vitamin C therapy (HIVC) resulted in a significant decrease in inflammatory markers compared to patients without HIVC [142].

Several meta-analyses are available which calculated the effect of especially high dose (mostly intravenous) vitamin C supplementation/therapy on clinical outcomes in (hospitalized) COVID-19 patients [90,143,144,145,146,147,148,149] (Table 2). 

While the studies up to December 2021 did not show an effect on mortality [90,143,144,145,146,149], the more recent ones did find a certain risk reducing effect of vitamin C [147,148].

However, Olczak-Pruc et al., for example, showed that the positive effects on mortality in hospital were only evident in randomized clinical trials and not in non-randomized ones, highlighting the importance of the study design [148]. Interestingly, the length of stay in the ICU was significantly longer in patients treated with vitamin C vs. standard therapy. Five of the six included studies for the meta-analysis showed a longer stay on average, with a pooled mean difference of 1.91 days (*p* < 0.001) [148]. One reason for this result might be reverse causality, meaning that patients with poor health might rather have received vitamin C therapy. In addition, possible bacterial infection secondary to the SARS-CoV-2 infection might prolong the stay in the ICU. Another reason might be side-effects of a high dose vitamin C therapy, although no adverse effects were reported in a previous meta-analysis of vitamin C supplementation in the critically ill [150].

Besides the dose, the timing of the administration of vitamin C seems to be important. Thus, positive outcomes might occur with early administration of vitamin C [151] to possibly prevent progression from mild to more severe COVID-19 [138,152].

Some populations are particularly affected by vitamin C deficiency, such as smokers who often have a lower vitamin C status and higher vitamin C requirements [153], and possibly also people who are exposed to increased oxidative stress such as alcoholics or those chronically exposed to severe air pollution [154]. Hyperglycemia or diabetes also promotes oxidative stress and a meta-analysis showed that the administration of vitamin C reduces glucose concentrations in type 2 diabetics [155]. Since patients suffering from diabetes and also smokers [156] are among the risk groups for worse COVID-19 outcomes, the prophylactic administration of lower doses of vitamin C could be useful in these risk populations [157]. This might be also valid for people with low consumption of fruits and vegetables. However, so far there is a lack of studies on the preventive effect of vitamin C on COVID-19 outcomes.

### 3.3. Zinc

Zinc, an almost omnipresent metal ion, is a vital trace element that plays an important role as a co-factor of numerous enzymes, such as those that exert important functions in DNA synthesis, cell growth and the defense against harmful reactive oxygen compounds [158,159]. Furthermore, zinc is essential for the immune system and regulates innate and adaptive immunity by influencing the proliferation and maturation of immune cells, and also acts as a modulator of immune responses and inflammation [159,160,161]. A zinc deficiency manifests itself in a disturbed function of both the innate and acquired immune systems, such as reduced chemotaxis and phagocytosis of polymorphonuclear leukocytes, leading to reduced NAPDH production and consequently reduced production of ROS for pathogen neutralization as well as reduced activity of natural killer cells and impaired function of T cells. Also affected by a lack of zinc is the ratio of memory versus naïve T and B cells [11,158,161]. 

Several studies have shown that zinc can inhibit viral replication (reviewed in [159,162]). The viruses studied were, for example, those causing the common cold [163], but also respiratory syncytial virus infections [164] and some forms of coronavirus [165]. A relevant observation is that the combination of Zn^2+^ ions and zinc-ionophores such as pyrithione at low concentrations (2 μM Zn^2+^ and 2 μM PT) can prevent the growth of SARS-coronavirus (SARS-CoV) in cell cultures [165].

Higher rates of inflammation as indicated by higher CRP and IL-6 levels are seen in patients with zinc deficiency. The decrease in plasma zinc during infections might also be part of an inflammatory response whereby hepatic metallothionein increases in response to inflammation and sequesters zinc in the liver [166]. In addition, the inflammatory response favors increased excretion of zinc, further lowering plasma zinc levels [159].

Several observational studies have provided evidence for an association between zinc status and COVID-19. In a French cross-sectional study, for example, it was shown that COVID-19 patients had a 2.4-fold higher prevalence of hypozincemia compared to non-COVID-19 participants. Particularly, older patients (>65 years) and medically assisted nursing home residents were at higher risk of hypozincemia. In addition, hypozincemia was shown as a prognostic factor for hospitalization for respiratory complications within 10 days (OR = 10.9, 95% CI: 2.3–51.6, *p* = 0.002) [167]. Heller et al. confirmed these findings. In their small cohort study of hospitalized patients, they showed that hypozincemia was more prevalent in COVID-19 patients, and occurred more frequently in the six non-survivors [168]. Likewise, in an observational cohort study in Spain of 249 COVID-19 patients with a median age of 65 years, serum zinc levels below 50 μg/dL at admission correlated with worse clinical presentation, longer time to recovery, and higher mortality compared to those with zinc concentrations higher than 50 μg/dL [169]. Finally, in another prospective study in hospitalized COVID-19 patients (n = 47), it was shown that SARS-CoV-2 positive patients had significantly lower zinc values compared to 45 healthy controls (median 74.5 μg/dL vs. 105.8 μg/dL) and more than half of the COVID-19 patients were found to be zinc deficient. The zinc deficient COVID-19 patients showed a 5.54 OR (95% CI 1.56–19.6) of developing complications [170]. 

Since zinc is important for the immune system and hypozincemia is convincingly prevalent in COVID-19 patients, several zinc supplementation trials in COVID-19 have been published to date and summarized in meta-analyses [90,171,172,173]. For example, Tabatabaeizadeh in his search up to September 2021 calculated an OR of 0.57 (95% CI: 0.43–0.77) for reduced mortality in the zinc intervention group in four studies with nearly 1400 participants [171]. In a more recent search up to August 2022 by Olczak-Pruc et al., an OR of 0.61 (95% CI: 0.35–1.06) for 28-day to 30-day mortality was calculated compared to patients who did not receive zinc [173]. In contrast, Beran et al. did not detect risk reduction through zinc supplementation in COVID-19 patients in their meta-analysis [90]. Different selected studies and/or pooling retrospective and RCTs results might be reasons for the differing results of the meta-analyses. In general, further RCTs are necessary to gain more insight into the beneficial effects of zinc supplementation in respiratory infectious diseases. 

A deficiency in zinc should be avoided, not only in pandemic times. Common risk groups are older people, and people with chronic diseases such as diabetes, liver disease, pathologies from the gastrointestinal tract, kidney diseases and also those with excess alcohol consumption [174]. An additional daily supplementation with low doses of zinc of the order of 5 to 10 mg, especially in periods where the immune system is strained, would make sense especially in the risk groups mentioned. Due to a lower supply and bioavailability, an additional risk group for zinc deficiency might be vegetarians/vegans who might also consider zinc supplementation at a low dose at least as long as the pandemic lasts. In cases of diagnosed zinc deficiency, higher therapeutic doses would be necessary to normalize the zinc status [174]. 

However, long-term chronic supplementation with high doses of zinc should be avoided; they may increase the risk of copper deficiency, in particular. Copper levels should be monitored while supplementing with larger amounts of zinc.

### 3.4. Selenium

Selenium is an essential trace element which, as a component of different enzymes/selenoproteins, notably the glutathione peroxidases but also thioredoxin reductases, protects in particular against oxidative stress. Moreover, as a component of the deiodinases, it has a second important function in thyroid metabolism. In addition, selenium is also important for immune function [175] and, for example, stimulates interferon production and the activity of natural killer cells [176]; therefore, selenium deficiency can affect host immunity and could alter virus virulence [177]. In this regard, there is good evidence that a low selenium status influences the host response to a number of RNA viruses leading to more severe clinical outcomes [178,179]. For example, in selenium deficient mice an influenza virus strain causing mild pneumonitis led to much more intensive pathogenicity [180]. 

Respiratory viruses such as SARS-CoV-2 promote the production of reactive oxygen compounds and thus disrupt the redox balance of the host [181,182]. In case of SARS-CoV-2, one mechanism discussed is a higher interaction of angiotensin II with its receptor AT1 (AT1R), due to lower bioavailability of ACE2. AT1R stimulation in turn mediates signals leading to an activation of NADPH oxidase and inducing oxidative stress and inflammatory response resulting in tissue damage. Moreover, NADPH oxidase-2 (NOX-2) is overexpressed in hospitalized COVID-19 patients [183]. 

Regarding the SARS-CoV-2 pandemic, Zhang et al. have identified a positive association between a higher cure rate from COVID-19 infection and adequate selenium status in 17 cities outside the region of Hubei (China) [184]. This effect is in line with the significant benefits of selenium supplementation shown against other viral infections [185]. Another study reported that COVID-19 patients who survived showed higher selenium serum levels than patients who died (53.3 ± 16.2 vs. 40.8 ± 8.1 µg/L) [186], and selenium status recovered with time in survivors while it remained low or even declined in non-survivors [186].

In a further report on 50 COVID-19 patients from Korea with and without pneumonia, 42% were selenium deficient [187]. Other studies also addressed the association between selenium status and COVID-19 outcomes (reviewed in [176]). In a systematic review with a search up to 27th of June 2021 including 11 studies with 681 COVID-19 patients and 164 healthy individuals, most of the studies showed lower selenium levels in patients compared to controls [188].

However, it is not definitely clear whether low selenium levels at baseline are a risk factor for COVID-19 or whether they are lowered during the disease. Regarding the latter hypothesis, for example, during systemic inflammatory conditions such as COVID-19, hepatic synthesis of selenoprotein P is diminished and plasma or serum selenium falls [168]. Recently, it was also shown that SARS-CoV-2 suppresses mRNA expression of different selenoproteins, such as glutathione peroxidase 4 (GPX4), in Vero cells [189]. By prevention of lipid peroxidation and ferroptotic cell death, GPX4 is important in the survival and expansion of recently activated T-cells [179,190]. There are thus some experimental data which suggest a possible decrease in functional selenoproteins during COVID-19. Whether this can be translated into higher selenium requirements during COVID-19 is not known. 

Optimizing selenium status may improve immune responses; however, selenium is distributed unevenly on the earth’s surface, leading to great variations in the selenium content among geological regions [191], which affect dietary selenium intake and could lead to selenium deficiency [192]. Although severe deficiency is rare, suboptimal selenium status is widespread [178,193]. Therefore, analyzing the selenium status of individuals, especially regions with low soil selenium content, could make sense in order to react with reasonable supplementation if necessary. At marginal selenium deficiency, this could be a dose of 50 μg of selenium daily especially in the flu season, in the elderly or in the context of a pandemic; this could help to improve immune responses, although possibly higher doses might be necessary to increase GPX activities [194].

However, selenium supplementation should be restricted to individuals with low or sub-optimal selenium intake or status since high supplemental doses and/or selenium status might have detrimental effects [185]. For example, in a randomized, double-blind, placebo-controlled study in adults aged fifty to 64 years with suboptimal selenium status, the effect of selenium supplementation on immune responses to flu vaccine was investigated. The results showed, among others, that although selenium stimulated T-cell proliferation (especially at doses of 100 µg/d) doses of 200 μg/d selenomethionine led to a reduction in granzyme positive CD8 cells [195]. High plasma levels and/or levels of exposure to selenium are also associated with an increased risk for type 2 diabetes [196], a well known risk factor for severe COVID-19 outcomes [197].

What should also be considered is that selenium blood levels decrease with age [176] and that mortality risk in elderly individuals with chronic age-associated diseases is increased by selenium deficiency [198]. This could have a relevance for worse COVID-19 outcomes in elderly patients with suboptimal selenium status.

### 3.5. Omega-3 Fatty Acids

Omega-3 fatty acids are polyunsaturated fatty acids (PUFAs) and their most important representatives are α-linolenic acid (ALA), eicosapentaenoic acid (EPA), and docosahexaenoic acid (DHA). The main source of EPA and DHA is fatty sea fish such as salmon, herring, and mackerel. Oils from microalgae contain DHA. ALA comes especially from flaxseed, walnuts, rape or their oils and can be converted to EPA and DHA, but the conversion rate of ALA to DHA is limited and depend on the demand for long chain polyunsaturated fatty acids and diet (elevated serum n-6/n-3 ratios—as with vegetarians—limit more strongly) [199].

EPA and DHA exert protective anti-inflammatory effects, potentially acting through the toll-like receptor 4 and G-protein coupled receptor 120 pathways to inhibit NF-κB and the connected inflammatory cascade [200] with cytokines such as IL-6 and TNF-α. They also upregulate glutathione (GSH), an antioxidant molecule, and inhibit lipid peroxidation [200,201]. In addition, they are metabolized to anti-inflammatory substances such as resolvins, protectins, and maresins [202].

Since the anti-inflammatory effects of omega-3 polyunsaturated fatty acids have been widely confirmed [203,204], supplementation studies with EPA and DHA have investigated the optimal dose, time for effective therapy and the potential for preventing inflammation. For example, Tan et al. demonstrated in a randomized controlled trial that high-dose n-3 PUFA supplementation (1.5 g/day EPA and 1.0 g/day DHA) considerably reduced plasma levels of IL-6, IL-1β and TNF-α after 4 and 8 weeks of therapy in 35 patients with chronic venous leg ulcers [205]. Likewise, daily supplementation of 2.4 g EPA + DHA over 8 weeks reduced inflammatory parameters in patients with obesity and diabetes [206]. In a recent umbrella review including 32 meta-analyses of intervention studies with amounts of supplemented EPA/DHA in the range of 600 mg to 3816 mg, it was shown that n-3 PUFA supplementation significantly reduced CRP, TNF-α and IL-6 concentrations [207].

Due to their anti-inflammatory effects, there is discussion as to whether omega-3 fatty acids could be beneficial regarding the “cytokine storm” in severe courses of COVID-19 [202,208,209]. For example, there is some meta-analytic evidence from 4 trials that omega-3 fatty acid supplementation in COVID-19 patients led to a significant reduction in CRP levels [210]. EPA and DHA also exert anti-thrombotic effects [211], which could also be beneficial in severe COVID-19 outcomes.

In this regard various human and animal studies have investigated the beneficial effects of EPA and DHA in patients with acute lung injury and ARDS, which also are clinical pathologies of severe COVID-19 [212,213]. Providing an immunomodulatory formula to enterally fed patients could optimize the therapeutic effect of fish oil. This was found in a meta-analysis in critically ill patients with ARDS, where omega-3 fatty acids in enteral nutrition formulas significantly improved pulmonary gas exchange and tended to shorten the length of stay in the ICU and duration of mechanical ventilation [214,215].

In another recent systematic review, it was shown that critically ill patients receiving parenteral nutrition therapy enriched with fish oil lipid emulsion showed a considerably reduced risk for infection (−40%, 24 RCTs) and sepsis (−56%, 9 RCTs) compared to standard parenteral nutrition. The mean length of ICU stay (10 RCTs) and the length of hospital stay (26 RCTs) were significantly reduced by about two days [216].

Regarding COVID-19, a few studies have addressed the potential of omega-3 fatty acid supplementation in treating COVID-19 (reviewed in [217]). By using a spike protein pseudo-virus, Goc et al. found that linolenic acid and EPA significantly block the entry of SARS-CoV-2, with EPA showing higher efficacy than linolenic acid in inhibiting the involved proteases TMPRSS2 and cathepsin L [218]. In this regard a double-blind, randomized clinical trial conducted in 128 critically ill patients infected with SARS-CoV-2 investigated the effects of supplementation with 1000 mg omega-3 daily containing 400 mg EPA and 200 mg DHA for 14 days. The intervention group showed a significantly higher 1-month survival rate and better acid-base parameters compared with the control group [219]. 

However, the administration of higher amounts of omega-3 fats could also be counterproductive, since “normal” inflammatory processes are important to activate the immune system. Based on singular experimental studies, it is estimated that at a dose corresponding to a human dose of 500 mg/d, omega-3 fatty acids exert beneficial effects over experimental bacterial infections, whereas 2–4 fold higher doses could have possibly negative effects [220]. Supplementation during an infection can also be detrimental if the balance between the needed inflammatory response and the anti-inflammatory effect is not achieved. For example, feeding fish oil to mice infected with an influenza virus made them more susceptible to the flu virus, with a higher mortality rate and longer recovery time [221]. The anti-inflammatory properties of supplementary high doses of fish oil may alter the immune response to influenza infection, at least in mice and at least in singular studies.

A good supply of omega-3-fatty acids might also positively affect mental health of COVID-19 patients. An umbrella review, including 14 meta-analyses, has shown that the prevalence of depression in SARS-CoV-2 infected patients ranged from 12% to 55% [222]. Lower doses of EPA (≤1 g/d) as EPA-pure or EPA-major (≥60% EPA) formulations might exert beneficial effects on depression [223].

In summary, an adequate supply of EPA and DHA in the range of 250 mg–500 mg/day and a correct ratio between alpha-linolenic acid and linoleic acids seems to be an additional important preventive strategy in the fight against viral infections.

## 4. Discussions and Recommendations

A healthy PBD with daily consumption of vegetables, fruits, legumes and also whole grains protects against severe courses of COVID-19 and over the long term reduces the risk for non-communicable diseases such as type 2 diabetes, cardiovascular diseases and some cancers [224,225,226], which are associated with worse outcomes of COVID-19. Meat and dairy products are important components of the diet, but to a much lesser extent than whole grains, fruits, vegetables, nuts and legumes. Fish is a healthy alternative to meat, especially as it provides EPA and DHA. Processed meat and also ultra-processed foods in general should be avoided. Despite the recommendation to focus on a PBD, the availability of food to meet energy and nutrient requirements in different regions of the world must be considered. In some parts of the world, for example, nutrient requirements can be met by vegetarian diets due to the varied food supply, while in other regions the lack of diversity of supply makes it necessary to eat meat as a source of nutrients.

Regarding nutrients, a good supply of vitamin D, vitamin C and zinc is especially important. Depending on the place of residence, skin pigmentation and age a daily supplementation of vitamin D of the order of 10–20 μg (400–800 IU) could be recommended especially in autumn and winter, although a higher dosage might be necessary to obtain the desired 25(OH)D blood levels.

Vitamin C is an important antioxidant, strengthens the immune system and can prevent inflammation. Vitamin C can be supplemented at a daily dose of 100 mg, especially when vegetable and fruit intake is low and also in smokers.

Zinc supports the body’s defense against infections. Due to its ubiquitous availability, the diet of most people provides sufficient zinc. Zinc at a daily dose of 5 to 10 mg could also be important during the COVID-19 crisis especially in risk groups such as vegetarians or vegans, who may have lower zinc bioavailability due to especially higher phytate intake.

In addition, other nutrients such as omega-3-fatty acids and selenium exert anti-inflammatory and/or antioxidative effects and should be provided in sufficient amounts. 

Previous studies on COVID-19 suggest that deficiencies in certain essential nutrients can impair the immune system and that correcting these deficiencies may be beneficial. However, studies also show that consuming any of these nutrients in excess of the body’s needs does not provide additional protection and may even be harmful. Therefore, it is important to be watchful for the at-risk groups and to check their nutritional status and consider it adequately.

Finally, it is important to keep one’s body weight within the normal range or to reduce overweight/obesity. Weight loss from a significant overweight status can be achieved through an energy-adjusted diet, limiting the consumption of foods particularly high in fat, and in low molecular weight carbohydrates, and increasing the intake of healthful plant-based foods. Recommended is a calorie reduction of 500–700 kcal less per day. However, even with mild symptoms that indicate COVID-19, the weight loss diet should be discontinued.

In addition to a high-quality diet, to support the immune system and reduce the risk of infection, a healthy lifestyle is recommended which includes not smoking [227], not drinking too much alcohol [228], regular physical activity based on health recommendations (e.g., typically in the range between 150 and 300 min per week at moderate intensity [229]), and good sleep hygiene [230].

Future studies could focus on topics such as nutrition as a personalized medicine in particular contexts. Examples could be oncological patients during pandemic times, as discussed in a recent review [231], and the effect of nutrition in the management of long-COVID-19 [232].

## Figures and Tables

**Table 1:** Vitamin D and COVID-19: Brief summary of selected meta-analyses regarding vitamin D supplementation or vitamin D status and SARS-CoV-2 infection risk or clinical outcomes of COVID-19 in adults *.

Title of Meta-Analysis Reference	Search Date Up to Study Number (Participants/Patients ^#^)	Major Results and/or Conclusions
Effects of vitamin D supplementation		
Vitamin D supplementation, COVID-19 and disease severity: a meta-analysis Shah K et al. [83]	up to December 2020 *n* = 3 (2 of which were randomized controlled trials, 1 retrospective case-control study; 532 patients)	ICU requirement—OR = 0.36; 95% CI: 0.210–0.626, mortality—OR: 0.93; 95% CI: 0.413–2.113.
The link between COVID-19 and VItamin D (VIVID): A systematic review and meta-analysis Bassatne A et al. [84]	up to 20 January 2021 *n* = 3	Conclusion: Calcifediol supplementation may have a protective effect on COVID-19 related ICU admissions. The current use of high doses of vitamin D in COVID-19 patients is not based on solid evidence.
The effect of vitamin D supplementation on mortality and intensive care unit admission of COVID-19 patients. A systematic review, meta-analysis and meta-regression Tentolouris N et al. [85]	up to 26 March 2021 *n* = 9 for mortality (2078 patients) *n* = 6 for ICU admission (860 patients)	Mortality—OR = 0.597; 95% CI: 0.318–1.121, ICU admission—OR = 0.326; 95% CI: 0.149–0.712.
Vitamin D and SARS-CoV-2 infection, severity and mortality: A systematic review and meta-analysis D’Ecclesiis O et al. [86]	up to April 2021 *n* = 6 (2 clinical trials, 2 cohort, 1 case-control and 1 cross-sectional studies)	Significant reduction of risk severity with supplemented vitamin D (SRR = 0.38, 95% CI: 0.20–0.72). Significant reduction in the risk of death with supplemented vitamin D (SRR = 0.35, 95% CI: 0.17–0.70).
Vitamin D supplementation and COVID-19 treatment: A systematic review and meta-analysis Rawat D et al. [87]	up to 18 May 2021 *n* = 5 (3 RCTs and 2 quasi-experimental studies; 467 patients)	Vitamin D did not reduce: mortality (RR = 0.55, 95% CI: 0.22–1.39) ICU admission rates (RR = 0.20, 95% CI: 0.01–4.26) need for invasive ventilation (RR = 0.24, 95% CI: 0.01–7.89).
COVID-19 and vitamin D (Co-VIVID study): a systematic review and meta-analysis of randomized controlled trials Varikasuvu SR et al. [88]	up to 5 August 2021 *n* = 6 (551 COVID-19 patients)	Vitamin D reduced overall COVID-19-related outcomes (RR = 0.60, 95% CI: 0.40–0.92).
Vitamin D supplementation for the treatment of COVID-19: A systematic review and meta-analysis of randomized controlled trials Kümmel LS et al. [89]	up to 17 September 2021 *n* = 8 RCTs (657 patients)	Mortality—OR = 0.74, 95% CI: 0.32–1.71. Stronger effects, when vitamin D was administered repeatedly (OR = 0.33, 95% CI: 0.1–1.14). ICU admission—OR = 0.41, 95% CI: 0.15–1.12, ICU admission and mechanical ventilation—OR = 0.52, 95% CI 0.27–1.02
Clinical significance of micronutrient supplements in patients with coronavirus disease 2019: A comprehensive systematic review and meta-analysis Beran A et al. [90]	up to 5 December 2021 *n* = 14 RCTs and observational studies (prospective and retrospective) (3497 patients)	Vitamin D did not reduce mortality (RR = 0.75, 95% CI: 0.49–1.17). Vitamin D reduced intubation rate (RR = 0.55, 95% CI: 0.32–0.97). Vitamin D reduced length of hospital stay (MD −1.26; 95% CI: −2.27–−0.25).
Effects of Vitamin D Supplementation on COVID-19 Related Outcomes: A Systematic Review and Meta-Analysis Hosseini B et al. [91]	up to January 2022 *n* = 5 on primary prevention (1 RCT, 4 NRISs) *n* = 5 on secondary prevention (2 RCTs, 3 NRISs) *n* = 13 on tertiary prevention (6 RCTs, 7 NRISs)	No significant effect on the risk of COVID-19 infection. A reduced risk of ICU admission (RR = 0.35, 95% CI: 0.20–0.62). A reduced risk of mortality (RR = 0.46, 95% CI: 0.30–0.70).
Hospital and laboratory outcomes of patients with COVID-19 who received vitamin D supplementation: a systematic review and meta-analysis of randomized controlled trials Zaazouee MS et al. [92]	up to July 2022 *n* = 9 (1586 COVID-19 patients)	ICU admission—RR = 0.59, 95% CI 0.41–0.84. Higher change in vitamin D level (standardized mean difference = 2.27, 95% CI: 2.08–2.47) compared to the control group. Other studied hospital and laboratory outcomes showed non-significant difference between vitamin D and the control group.
Protective Effect of Vitamin D Supplementation on COVID-19-Related Intensive Care Hospitalization and Mortality: Definitive Evidence from Meta-Analysis and Trial Sequential Analysis Argano C et al. [93]	up to 20 September 2022 *n*= 5 RCTs (total number of patients not indicated)	Vitamin D administration results in a decreased risk of death and ICU admission (standardized mean difference, 95% CI: 0.49, 0.34–0.72, and 0.28, 0.20–0.39, respectively).
Association of vitamin D status with different outcomes		
Vitamin D insufficiency as a potential culprit in critical COVID-19 patients Munshi R et al. [94]	up to 8 June 2020 *n* = 6 (376 patients)	Patients with poor prognosis had significantly lower serum levels of vitamin D compared with those with good prognosis MD = −0.58 (95% Cl: −0.83 to −0.34).
Low vitamin D status is associated with coronavirus disease 2019 outcomes: a systematic review and meta-analysis Liu NY et al. [95]	up to 25 September 2020 *n* = 10 (case-control studies; 361,934 participants)	Vitamin D deficiency or insufficiency was associated with an increased risk of COVID-19 (OR = 1.43, 95% CI: 1.00–2.05). COVID-19-positive individuals had lower vitamin D levels than COVID-19-negative individuals (SMD = −0.37, 95% CI: −0.52 to −0.21).
Vitamin D deficiency aggravates COVID-19: systematic review and meta-analysis Pereira M et al. [96] Addendum to vitamin D deficiency aggravates COVID-19: systematic review and meta-analysis Damascena AD et al.	up to 9 October 2020 *n* = 27 (372,332 participants) *n* = 22	Vitamin D deficiency was not associated with a higher chance of infection by COVID-19 (OR = 1.35; 95% CI: 0.80–1.88). Severe cases of COVID-19 showed more vitamin D deficiency compared with mild cases (OR = 1.64; 95% CI: 1.30–2.09). Vitamin D insufficiency increased hospitalization (OR = 1.81, 95% CI: 1.41–2.21) and mortality from COVID-19 (OR = 1.82, 95% CI: 1.06–2.58). Addendum/Correction: After updating the study conclusions remained unchanged.
Association of Vitamin D Status with SARS-CoV-2 Infection or COVID-19 Severity: A Systematic Review and Meta-analysis Kazemi A. et al. [97]	up to 26 November 2020 *n* = 39 (total number of participants not indicated)	Vitamin D deficiency was associated with a higher risk of SARS-CoV-2 infection (OR = 1.77; 95% CI: 1.24–2.53), composite severity (OR = 2.57; 95% CI: 1.65–4.01). No relation was observed (OR: 1.05; 95% CI: 0.63–1.75) with mortality in adjusted studies that used logistic regression. ICU admission showed inconsistent results.
The relationship between the severity and mortality of SARS-CoV-2 infection and 25-hydroxyvitamin D concentration—a meta-analysis Oscanoa TJ et al. [98]	up to December 2020 *n* = 23 (5 cohort, 11 cases and controls, 7 cross sectional observational studies; 2692 participants)	Vitamin D deficiency was associated with increased risk of severe SARS-CoV-2 disease (RR = 2.00; 95% CI: 1.47–2.71) and mortality (RR = 2.45; 95% CI: 1.24–4.84).
Association of vitamin D deficiency with COVID-19 infection severity: Systematic review and meta-analysis Wang Z et al. [99]	up to 3 December 2020 *n* = 17 observational studies (2756 patients)	Vitamin D deficiency was associated with significantly higher mortality—OR = 2.47, 95% CI: 1.50–4.05, higher rates of hospital admissions—OR = 2.18, 95% CI: 1.48–3.21, longer hospital stays (0.52 days; 95% CI: 0.25–0.80; 2 studies) as compared to non-vitamin D deficient status.
Low Serum 25-hydroxyvitamin D (Vitamin D) Level Is Associated With Susceptibility to COVID-19, Severity, and Mortality: A Systematic Review and Meta-Analysis Akbar MR et al. [100]	up to 9 December 2020 *n* = 14 (999,179 participants)	Low serum 25(OH)D was associated with higher rate of COVID-19 infection compared to the control group (OR = 2.71, 95% CI: 1.72–4.29). Higher rate of severe COVID-19 was observed in patients with low serum 25(OH)D (OR = 1.90, 95% CI: 1.24, 2.93). Low serum 25(OH)D was associated with higher mortality (OR = 3.08, 95% CI: 1.35, 7.00).
The role of vitamin D deficiency on COVID-19: a systematic review and meta-analysis of observational studies Kaya MO et al. [101]	up to 15 December 2020 *n* = 21 (205,869 participants)	Individuals with low serum vitamin D levels were 1.64 times (95% CI: 1.32–2.04) more likely to contract COVID-19. Individuals with 25(OH)D levels below 20 ng/mL (50 nmol/L) were 2.42 times (95% CI: 1.13–5.18) more likely to have severe COVID-19. Low vitamin D levels had no effect on COVID-19 mortality (OR = 1.64; 95% CI: 0.53–5.06).
The role of vitamin D in the age of COVID-19: A systematic review and meta-analysis Ghasemian R et al. [102]	up to 18 December 2020 *n* = 23 (11,901 participants)	SARS-CoV-2 infection risk in individuals with vitamin D deficiency (OR = 3.3, 95% CI: 2.5–4.3). Severe stages of COVID-19 risk in patients with vitamin D deficiency (OR = 5.1, 95% CI: 2.6–10.3). No significant association between vitamin D deficiency and higher mortality rates (OR = 1.6, 95% CI: 0.5–4.4).
The link between COVID-19 and VItamin D (VIVID): A systematic review and meta-analysis Bassatne A et al. [84]	up to 18 December 2020 *n* = 31 observational studies (total number of participants not indicated)	A positive (not significant) trend between serum 25(OH)D level < 20 ng/mL and an increased risk of mortality, ICU admission, invasive ventilation, non-invasive ventilation or SARS-CoV-2 positivity. Mean 25(OH)D levels were 5.9 ng/mL (95% CI −9.5 to −2.3) significantly lower in COVID-19 positive, compared to negative patients.
The Impact of Vitamin D Level on COVID-19 Infection: Systematic Review and Meta-Analysis Teshome A et al. [103]	up to 20 December 2020 *n* = 14 (cohort studies, case-control studies, cross-sectional studies and interim audit, 91,120 participants)	Vitamin D deficiency (OR = 1.80, 95% CI: 1.72–1.88) for COVID-19 infection as compared to participants with sufficient Vitamin D levels.
The Impact of Vitamin D Deficiency on the Severity of Symptoms and Mortality Rate among Adult Patients with COVID-19: A Systematic Review and Meta-Analysis Al Kiyumi MH et al. [104]	up to 20 December 2020 *n* = 43 (254,963 patients)	Lower vitamin D levels correlate with severity of symptoms (OR = 3.38, 95% CI: 1.94–5.87), case fatality rate (OR = 2.30, 95% CI: 1.47–3.59).
Influence of 25-hydroxy-cholecalciferol levels on SARS-CoV-2 infection and COVID-19 severity: A systematic review and meta-analysis Crafa A et al. [105]	up to January 2021 *n* = 30 (total number of participants not indicated)	Serum levels of 25(OH)D were significantly lower in patients with SARS-CoV-2 infection than in negative ones MD −3.99 (−5.34, −2.64), in patients with severe disease MD −6.88 (−9.74, −4.03), and in those who died of COVID-19 MD −8.01 (−12.50, −3.51). Vitamin D deficient patients had an increased risk of developing severe disease (OR = 4.58, 95% CI: 2.24–9.35) but not a fatal outcome (OR = 4.92, 95% CI: 0.83–29.31). After updating the study conclusions remained unchanged.
Therapeutic and prognostic role of vitamin D for COVID-19 infection: A systematic review and meta-analysis of 43 observational studies Petrelli F et al. [106]	up to 31 January 2021 *n* = 43 observational studies (612,601 participants)	Risk of COVID-19 infection higher in vitamin D deficiency (OR = 1.26; 95% CI: 1.19–1.34). Vitamin D deficiency was also associated with worse severity—OR = 2.6; 95% CI: 1.84–3.67, higher mortality—OR = 1.22; 95% CI: 1.04–1.43.
COVID-19 Mortality Risk Correlates Inversely with Vitamin D3 Status, and a Mortality Rate Close to Zero Could Theoretically Be Achieved at 50 ng/mL 25(OH)D3: Results of a Systematic Review and Meta-Analysis Borsche L et al. [107]	up to 27 March 2021 *n* = 8 (one population study and seven clinical studies)	Reported vitamin D3 blood levels pre-infection or on the day of hospital admission analyzed independently showed a negative Pearson correlation of vitamin D3 levels and mortality risk (r(17) = −0.4154, *p* = 0.0770/r(13) = −0.4886, *p* = 0.0646). For the combined data a significant Pearson correlation was observed (r(32) = −0.3989, *p* = 0.0194).
Association between Vitamin D Status and Risk of Developing Severe COVID-19 Infection: A Meta-Analysis of Observational Studies Ben-Eltriki M et al. [108]	up to 30 March 2021 *n* = 24 observational studies (3637 participants)	Low vitamin D status was associated with higher risk of death (RR = 1.60, 95% CI: 1.10–2.32), higher risk of developing severe COVID-19 pneumonia (RR = 1.50, 95% CI: 1.10–2.05).
Vitamin D Status and SARS-CoV-2 Infection and COVID-19 Clinical Outcomes Chiodini I et al. [109]	up to 31 March 2021 *n* = 54 (49 as fully-printed and 5 as pre-print publications; 1,403,715 individuals)	Severe deficiency, deficiency and insufficiency of vitamin D were all associated with ICU admission (OR, 95% Cis: 2.63, 1.45–4.77; 2.16, 1.43–3.26; 2.83, 1.74–4.61, respectively), mortality (OR, 95% CIs: 2.60, 1.93–3.49; 1.84, 1.26–2.69; 4.15, 1.76–9.77, respectively), SARS-CoV-2 infection (OR, 95% Cis: 1.68, 1.32–2.13; 1.83, 1.43–2.33; 1.49, 1.16–1.91, respectively), COVID-19 hospitalization (OR, 95% CIs: 2.51, 1.63–3.85; 2.38, 1.56–3.63; 1.82, 1.43–2.33).
Vitamin D and SARS-CoV2 infection, severity and mortality: A systematic review and meta-analysis D’Ecclesiis O et al. [86]	up to April 2021 *n* = 38 (205,565 patients)	Higher infection risk with low serum vitamin D levels compared to the highest level: SRR = 2.18 (95% CI: 1.55–3.06). Increased risk of severity with low serum 25(OH)D levels (SRR = 2.38, 95% CI: 1.53–3.70). Increased risk of death with low levels of 25(OH)D (SRR = 2.35, 95% CI: 1.46–3.80).
Effects of Vitamin D Serum Level on Morbidity and Mortality in Patients with COVID-19: A Systematic Review and Meta-Analysis Hu Y et al. [110]	up to 1 May 2021 *n* = 20 observational studies (12,806 patients)	Mortality—RR = 1.49 (95% CI: 1.34–1.65). ICU admission—RR = 0.87 (95% CI: 0.67–1.14). Ventilator support—RR = 1.29 (95% CI: 0.79–1.84). Length of hospital stay—RR = 0.84 (95% CI -0.45 to 2.13).
A systematic review and meta-analysis of effect of vitamin D levels on the incidence of COVID-19 Szarpak L et al. [111]	up to 10 May 2021 *n* = 13 (14,485 participants)	Mean vitamin D level in SARS-CoV-2 negative patients was 17.7 ± 6.9 ng/mL compared to SARS-CoV-2 positive patients 14.1 ± 8.2 ng/mL (MD = 3.93; 95% CI: 2.84–5.02).
Prognostic and Therapeutic Role of Vitamin D in COVID-19: Systematic Review and Meta-analysis Dissanayake HA et al. [112]	up to 30 May 2021 *n* = 72 observational studies (1,976,099 individuals)	1. Vitamin D deficiency/insufficiency increased the odds of developing COVID-19 (OR = 1.46; 95% CI: 1.28–1.65), severe disease (OR = 1.90; 95% CI: 1.52–2.38), death (OR = 2.07; 95% CI: 1.28–3.35). 2. 25(OH)D concentrations were lower in individuals with COVID-19 compared with controls (MD = −3.85 ng/mL; 95% CI: −5.44 to −2.26), in patients with severe COVID-19 compared with controls with nonsevere COVID-19 (MD = −4.84 ng/mL; 95% CI: −7.32 to −2.35), in nonsurvivors compared with survivors (MD = −4.80 ng/mL; 95% CI: −7.89 to −1.71). 3. The association between vitamin D deficiency/insufficiency and death was insignificant when studies with high risk of bias or studies reporting unadjusted effect estimates were excluded.
Low vitamin D levels do not aggravate COVID-19 risk or death, and vitamin D supplementation does not improve outcomes in hospitalized patients with COVID-19: a meta-analysis and GRADE assessment of cohort studies and RCTs Chen J et al. [113]	up to 5 June 2021 *n* = 11 cohort studies (536,105 patients)	1. Vitamin D deficiency (<20 ng/mL) or insufficiency (<30 ng/mL) was not associated with a significant increased risk of COVID-19 infection (OR for <20 ng/mL = 1.61, 95% CI: 0.92–2.80) or in-hospital death (OR for <20 ng/mL = 2.18, 95% CI: 0.91–5.26; OR for <30 ng/mL = 3.07, 95% CI: 0.64–14.78). 2. Each 10 ng/mL increase in serum vitamin D was not associated with a significant decreased risk of COVID-19 infection (OR = 0.92, 95% CI: 0.79–1.08) or death (OR = 0.65, 95% CI: 0.40–1.06).
Association between vitamin D status and risk of COVID-19 in-hospital mortality: A systematic review and meta-analysis of observational studies Ebrahimzadeh A et al. [114]	up to 27 July 2021 *n* = 13 observational studies	A significant positive relationship was found between vitamin D deficiency and risk of COVID-19 in-hospital mortality (OR = 2.11; 95% CI: 1.03–4.32). An inverse significant association was found between each unit increment in serum vitamin D concentrations and risk of COVID-19 in-hospital mortality (OR = 0.94; 95% CI: 0.89, 0.99).
Vitamin D Deficiency and Comorbidities as Risk Factors of COVID-19 Infection: A Systematic Review and Meta-analysis Mishra P et al. [115]	up to 20 August 2021 *n* = 16 observational cohort and case-control studies (386,631 patients)	Significantly lower vitamin D levels in COVID-19 positive patients (MD, −1.70; 95% CI: −2.74 to −0.66). Male patients showed higher odds of having low vitamin D levels (OR = 2.09; 95% CI: 1.38 to 3.17) than female patients (OR = 1.17; 95% CI: 0.74 to 1.86; *p* = 0.477).

* published until 20 January 2023, searched in PUBMED. Exclusion criteria were age <18 y and pregnancy. The sort order of the papers according to the search date. SRR: summary relative risk. ^#^ Total number of participants/patients included in the systematic review/meta-analysis.

**Table 2:** Vitamin C and COVID-19: Brief summary of selected meta-analyses of trials studying the effect of vitamin C treatment on clinical outcomes in COVID-19 patients *.

Title of Meta-Analysis Reference	Search Date Up to Study Number (Participants/Patients ^#^)	Major Results and/or Conclusions
Intravenous vitamin C use and risk of severity and mortality in COVID-19: A systematic review and meta-analysis Ao GY et al. [143]	up to 23 June 2021 *n* = 7	Intravenous vitamin C treatment compared with placebo treatment or usual care did not significantly affect disease severity, OR = 0.70; 95% CI: 0.45–1.07 mortality, OR = 0.64; 95% CI: 0.41–1.00.
Association of Vitamin C Treatment with Clinical Outcomes for COVID-19 Patients: A Systematic Review and Meta-Analysis Huang WY et al. [144]	up to June 2022 *n* = 19 (2765 participants)	The intervention group tended to have a lower risk ratio in all-cause mortality (RR = 0.81, 95% CI: 0.62 to 1.07). There were no significant differences in ventilation incidence, hospitalization duration, and length of ICU stay between the two groups.
The effectiveness of high-dose intravenous vitamin C for patients with coronavirus disease 2019: A systematic review and meta-analysis Kwak SG et al. [145]	up to 29 July 2021 *n* = 5	In-hospital mortality rate was not significantly different between the high-dose intravenous vitamin C intervention and control groups (OR = 0.551, 95% CI: 0.290–1.047). The length of hospital stay was also not significantly different.
Vitamin C and COVID-19 treatment: A systematic review and meta-analysis of randomized controlled trials Rawat D et al. [149]	up to 18 September 2021 *n* = 6 (572 patients)	Vitamin C treatment did not reduce: mortality (RR = 0.73, 95% CI: 0.42 to 1.27), ICU length of stay (SMD = 0.29, 95% CI: −0.05 to 0.63), hospital length of stay (SMD = −0.23, 95% CI: −1.04 to 0.58), need for invasive mechanical ventilation (Risk Ratio = 0.93, 95% CI: 0.61 to 1.44).
Clinical significance of micronutrient supplements in patients with coronavirus disease 2019: A comprehensive systematic review and meta-analysis Beran A. et al. [90]	up to 5 December 2021 *n* = 9 (1488 patients)	Vitamin C supplementation had no significant effect on: mortality (RR = 1.00, 95% CI: 0.62–1.62), intubation rate (RR = 1.77, 95% CI: 0.56–5.56), length of hospital stay (MD 0.64; 95% CI: −1.70–2.99).
Effect of Vitamin C on Clinical Outcomes of Critically Ill Patients With COVID-19: An Observational Study and Subsequent Meta-Analysis Gavrielatou E et al. [146]	up to 18 December 2021 *n* = 11 (6 observational; five randomized controlled trials; 1807 patients)	Mortality of patients receiving vitamin C on top of standard-of-care was not lower than patients receiving standard-of-care alone (25.8 vs. 34.7%; RR = 0.85, 95% CI: 0.57–1.26).
Impact of high-dose vitamin C on the mortality, severity, and duration of hospital stay in COVID-19 patients: A meta-analysis Bhowmik KK et al. [147]	up to 30 May 2022 *n* = 15 (2125 participants)	Vitamin C significantly reduced mortality risk in COVID-19 patients (OR = 0.54, 95% CI: 0.42–0.69). Vitamin C showed 0.63 times less severity (OR = 0.63, 95% CI: 0.43–0.94). Vitamin C treatment led to slightly longer stay in hospital (MD = 0.19, 95% CI: −1.57 to 1.96).
Vitamin C Supplementation for the Treatment of COVID-19: A Systematic Review and Meta-Analysis Olczak-Pruc M et al. [148]	up to 28 August 2022 *n* = 19	In-hospital mortality with and without vitamin C supplementation was 24.1% vs. 33.9% (OR = 0.59; 95% CI: 0.37–0.95). In randomized clinical trials, in-hospital mortality varied and amounted to 23.9% vs. 35.8% (OR = 0.44; 95% CI: 0.25 to 0.76). In non-randomized trials, in-hospital mortality was not significantly different. In intravenous vitamin C supplementation, in-hospital mortality was not significant. The ICU length of stay was longer in patients treated with vitamin C vs. standard therapy, (MD = 1.91; 95% CI: 0.89–2.93). Acute kidney injury in patients treated with and without vitamin C varied and amounted to 27.8% vs. 45.0% (OR = 0.56; 95% CI: 0.40–0.78).

* Exclusion criteria were age <18 y and pregnancy. Published until 23 January 2023; searched in PUBMED. Sort order according to the search date. ^#^ Total number of participants/patients included in the systematic review/meta-analysis.

## Data Availability

Not applicable.
